# Risk factors for postoperative venous thromboembolism in patients with lung cancer: a systematic review and meta-analysis

**DOI:** 10.3389/fmed.2025.1699892

**Published:** 2026-01-14

**Authors:** Jie Fu, Yiyi Zhou, Feng Zhang, Ru Lv, Lu Hu, Haiyan Zhang

**Affiliations:** 1 Department of Cardiology, Army Medical Center of PLA, Chongqing, China; 2 Department of Otolaryngology, Army Medical Center of PLA, Chongqing, China; 3 Department of Oncology, Army Medical Center of PLA, Chongqing, China

**Keywords:** lung cancer, meta-analysis, postoperative complications, risk factor, venous thromboembolism

## Abstract

**Objective:**

Venous thromboembolism (VTE) is a serious complication following lung cancer surgery, which not only complicates treatment but may also delay cancer-specific therapies and even threaten patient survival. Currently, the risk factors for postoperative VTE in lung cancer patients remain unclear. Therefore, we conducted a meta-analysis to identify risk factors associated with VTE in these patients after surgery.

**Methods:**

We systematically searched PubMed, Embase, Web of Science, Cochrane Library, China National Knowledge Infrastructure (CNKI), Wanfang Database, Chinese Biomedical Literature Database (CBM), and VIP Database for studies investigating risk factors for VTE after lung cancer surgery. The search covered the period from database inception to February 2025. Two reviewers independently screened the literature based on the inclusion and exclusion criteria, extracted data, and assessed the risk of bias in the included studies. Meta-analysis was performed using RevMan 5.4 software.

**Results:**

A total of 21 studies involving 41,780 participants were included. The meta-analysis identified the following significant risk factors for VTE after lung cancer surgery: age ≥ 65 years old, hyperlipidemia, tumor staging III–IV, thoracotomy, operation time ≥ 2 h, intraoperative blood loss ≥ 200 mL, abnormal D-dimer levels, and preoperative chemotherapy. In contrast, no statistically significant associations were found between VTE occurrence and sex, age ≥ 60 years, smoking history, drinking history, body mass index ≥ 25 kg/m^2^, hypertension, coronary heart disease, diabetes, pathological type, operation time ≥ 3 h, tumor location, or type of lung resection.

**Conclusion:**

This meta-analysis confirmed that age ≥ 65 years, hyperlipidemia, advanced tumor stage (III–IV), thoracotomy, prolonged operation time (≥ 2 h), significant intraoperative blood loss (≥ 200 mL), abnormal D-dimer, and preoperative chemotherapy were risk factors for VTE in lung cancer patients after surgery. Targeted preventive measures based on these factors may help improve clinical outcomes in this patient population.

## 1 Introduction

Lung cancer remains one of the most prevalent malignancies worldwide, with its incidence and mortality rates consistently ranking first among all cancers (1–5). For eligible patients, comprehensive treatment centered around surgery remains the primary clinical approach, as it effectively removes lesion tissues and improves survival outcomes (6, 7). However, surgical trauma in lung cancer patients can lead to coagulation dysfunction, resulting in a hypercoagulable state and altered hemorheology. Postoperative pain further impedes early mobilization (8, 9), collectively contributing to a high susceptibility to venous thromboembolism (VTE) after surgery (10). Studies have reported that the incidence of postoperative VTE in lung cancer patients ranges from approximately 7.3% to 13.9% (11). As a serious complication following lung cancer surgery, VTE not only complicates clinical management but may also delay cancer-specific treatment and even threaten patient survival (12). Therefore, identifying risk factors for VTE in these patients is crucial for improving prognosis. Although multiple studies have investigated these risk factors, their findings remain inconsistent (13–33). This study aims to evaluate the risk factors for VTE after lung cancer surgery through a meta-analysis, thereby providing evidence-based support for postoperative VTE prevention.

## 2 Methods

The study was conducted and reported in accordance with the Preferred Reporting Items for Systematic Reviews and Meta-Analyses (PRISMA) guidelines (34).

### 2.1 Literature search

A systematic literature search was performed across the following electronic databases: PubMed, Embase, Web of Science, Cochrane Library, China National Knowledge Infrastructure (CNKI), Wanfang Database, the China Biomedical Literature Database (CBM), and VIP Database. The search period spanned from the inception of each database to February 2025 to identify all relevant studies investigating risk factors for VTE following lung cancer surgery. The search strategy combined Medical Subject Headings (MeSH) terms with free words, including but not limited to “lung cancer,” “venous thromboembolism,” “deep vein thrombosis,” “pulmonary embolism,” and “risk factor.” The specific search strategy used for PubMed is provided as an example in Supplementary Table S1.

### 2.2 Inclusion and exclusion criteria

The study eligibility criteria were defined as follows:

Inclusion criteria:

1. Participants: Patients aged 18 years or older who were pathologically diagnosed with lung cancer and underwent surgical resection;
2. Exposure: Investigation of risk factors for postoperative VTE;
3. Outcome: A clear diagnosis of VTE confirmed by imaging examinations;
4. Study design: Cohort or case–control studies.

Exclusion criteria:

1. Duplicate publication;
2. Studies published as case reports, conference abstracts, animal studies, reviews, etc.;
3. Publications with insufficient data for extraction;
4. Studies with a Newcastle–Ottawa Scale (NOS) score below 5 points.

### 2.3 Data extraction

Two investigators independently screened the retrieved literature, extracted data, and cross-checked their findings. Any disagreements were resolved through discussion until a consensus was reached. The extracted information included the first author, publication year, study design, sample size, VTE incidence, exposure factors examined, and reported outcomes.

### 2.4 Risk of bias assessment

The methodological quality and risk of bias of the included studies were assessed independently by two reviewers using the NOS. The NOS evaluates studies based on three domains: selection of study groups, comparability of groups, and ascertainment of either exposure or outcome. The total score ranges from 0 to 9 points. Studies were categorized as low (0–4 points), moderate (5–6 points), or high quality (7–9 points). Consistent with the exclusion criteria, only studies with a NOS score of 5 or higher were included in the final meta-analysis.

### 2.5 Statistical analysis

All meta-analyses were performed using RevMan software (version 5.4). For consistency, all outcome data were converted into odds ratios (ORs) with their corresponding 95% confidence intervals (CIs). Pooled ORs and 95% CIs were calculated for each risk factor. Heterogeneity across included studies was assessed using chi-square tests and quantified by the *I^2^* statistic. A fixed-effects model was used when no significant heterogeneity was present (*p* ≥ 0.10 and *I^2^* ≤ 50%); otherwise, a random-effects model was applied. Sensitivity analyses were conducted by alternating between the fixed- and random-effects models to evaluate the robustness of the pooled results. Publication bias was assessed using Egger’s test or funnel plots for risk factors that were reported in 10 or more studies.

## 3 Results

### 3.1 Literature retrieval results

**Figure 1:**
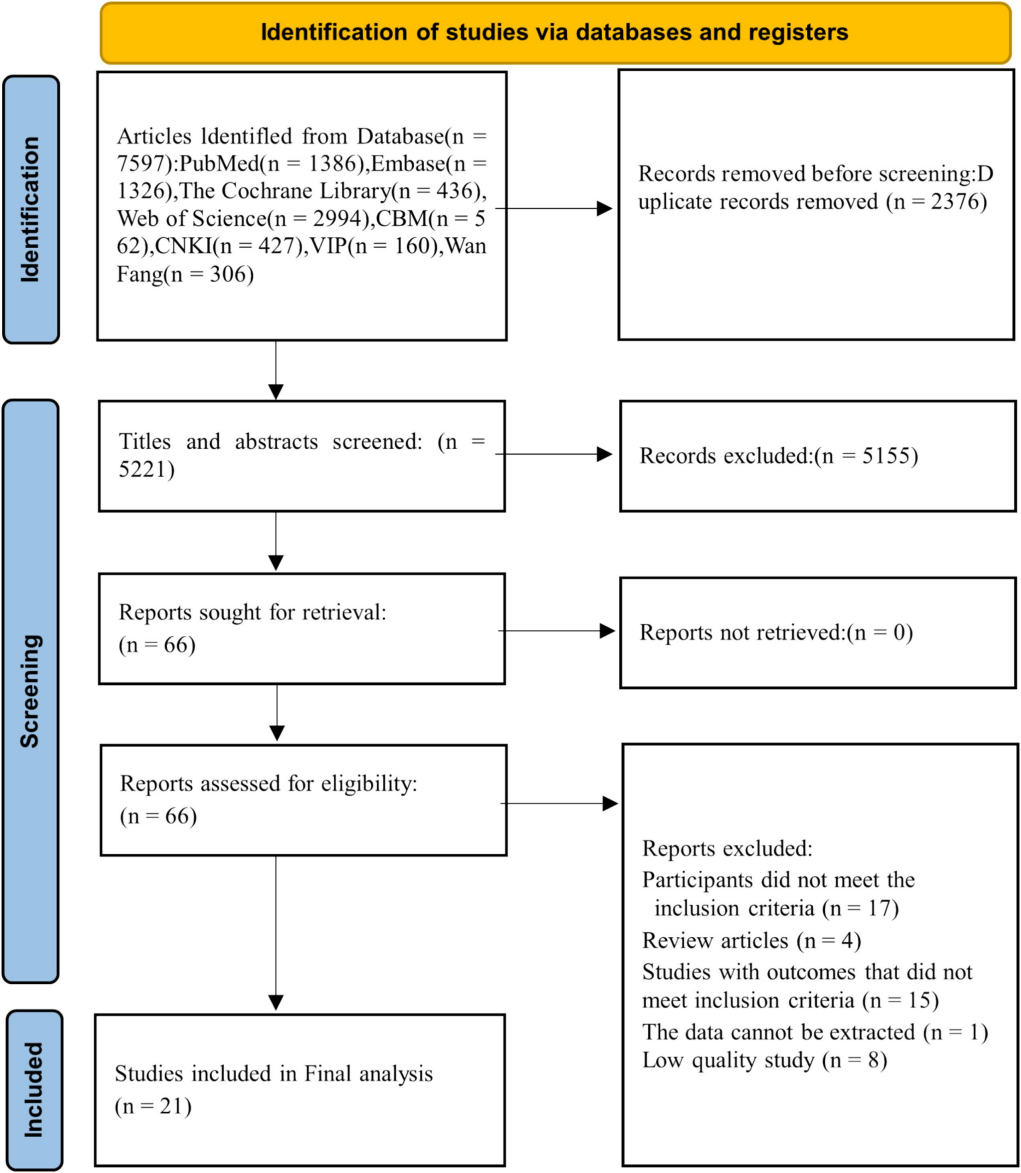
Flowchart of literature selection.

The initial systematic search identified 7,597 potentially relevant records. Following a rigorous screening process of titles, abstracts, and full texts against the predefined inclusion and exclusion criteria, 21 studies (13–33) were ultimately included for meta-analysis (Figure 1). The included studies, conducted in the United States, China, and Canada and published between 2012 and 2024, comprised both case–control and cohort designs. Sample sizes ranged from 84 to 14,308 participants. The methodological quality assessed by the NOS was high, with scores ranging from 7 to 9. The VTE incidence rate across studies was 3.63%. The baseline characteristics of the included studies are summarized in Table 1.

**Table 1:** Characteristics of the included studies.

Study, year	Country	Study design	Type of VTE	Sample size	Number of VTE cases	Male,n(%)	Age(Mean)	NOS score	Risk factor
Akhtar-Danesh et al. (24)	Canada	Case–control study	VTE	12,626	345	5,923(46.91%)	NR	9	①②⑥⑧⑨⑩⑪
Awang et al. (20)	China	Case–control study	DVT	108	36	64(59.26%)	65.7	9	①⑤⑧⑩⑪⑫⑬⑰
Cui et al. (18)	China	Case–control study	VTE	339	39	166(48.98%)	60.1	8	①③⑥⑧⑨⑩⑪⑫⑮
Ding et al. (30)	China	Case–control study	VTE	601	63	289(48.09%)	61	9	①③⑤⑧⑩⑪⑫⑯⑰
Dong et al. (23)	China	Case–control study	VTE	132	11	42(31.82%)	51	8	①③④⑥⑦⑨
Dong et al. (27)	China	Cohort study	VTE	314	23	117(37.26%)	57	7	①③④⑥⑦⑧⑨⑩⑪⑫⑮⑯⑱
Du et al. (25)	China	Case–control study	DVT	83	25	51(61.45%)	NR	7	①②③⑤⑥⑧⑩⑪⑫⑮
Hei et al. (33)	China	Case–control study	PE	90	45	54(60.00%)	58.6	7	①③④⑥⑧⑩⑪⑬⑭
Jia et al. (17)	China	Case–control study	DVT	403	54	262(65.01%)	59	8	①③④⑧⑩⑪⑬⑭⑱
Ke et al. (16)	China	Case–control study	VTE	160	43	73(45.63%)	NR	8	①②③④⑤⑥⑨⑬
Li et al. (32)	China	Cohort study	PE	9,726	55	5,326(54.76%)	66.5	7	①⑧⑩⑪⑫⑯
Qiao et al. (22)	China	Case–control study	DVT	222	74	139(62.61%)	60.04	8	①⑧⑩⑪⑫⑯⑰
Qin (15)	China	Case–control study	VTE	227	63	85(37.44%)	56.99	7	①③④⑥⑧⑩⑪⑮
Qin et al. (28)	China	Case–control study	VTE	502	138	171(34.06%)	56.99	7	③④⑥
Song et al. (29)	China	Cohort study	VTE	262	30	149(56.87%)	54.73	8	①⑤⑥⑫⑯
Thomas et al. (31)	America	Case–control study	VTE	14,308	234	6,630(46.34%)	NR	9	①②③⑤⑥⑫⑯
Wang (14)	China	Case–control study	VTE	354	50	188(53.11%)	62.4	7	①③⑥⑧⑨⑩⑪⑫
Wu et al. (13)	China	Case–control study	VTE	84	18	46(54.76%)	60.75	7	①②③④⑥⑧⑨⑩⑪⑰⑱
Yang et al. (26)	China	Case–control study	VTE	1,001	53	656(65.53%)	NR	7	①②⑧⑩⑪⑰⑱
Zhang et al. (19)	China	Case–control study	VTE	118	59	74(62.71%)	61.5	8	①⑥⑧⑨⑮
Zhou et al. (21)	China	Case–control study	DVT	120	60	76(63.33%)	70.07	7	①⑤⑦⑬

① Sex; ② Age; ③ Smoking history; ④ Drinking history; ⑤ Body mass index; ⑥ Hypertension; ⑦ Hyperlipidemia; ⑧ Tumor stage; ⑨ Coronary heart disease; ⑩ Diabetes; ⑪ Pathological types; ⑫ Surgical approach; ⑬ Operative time; ⑭ Intraoperative bleeding; ⑮ Tumor site; ⑯ Type of lung resection; ⑰ History of chemotherapy; ⑱ D-dimer; VTE, Venous thromboembolism; PE, Pulmonary embolism; DVT, Deep vein thrombosis; NOS, Newcastle–Ottawa scale; NR, Not reported.

### 3.2 Meta-analysis results

#### 3.2.1 Patient factors

##### 3.2.1.1 Sex

**Figure 2:**
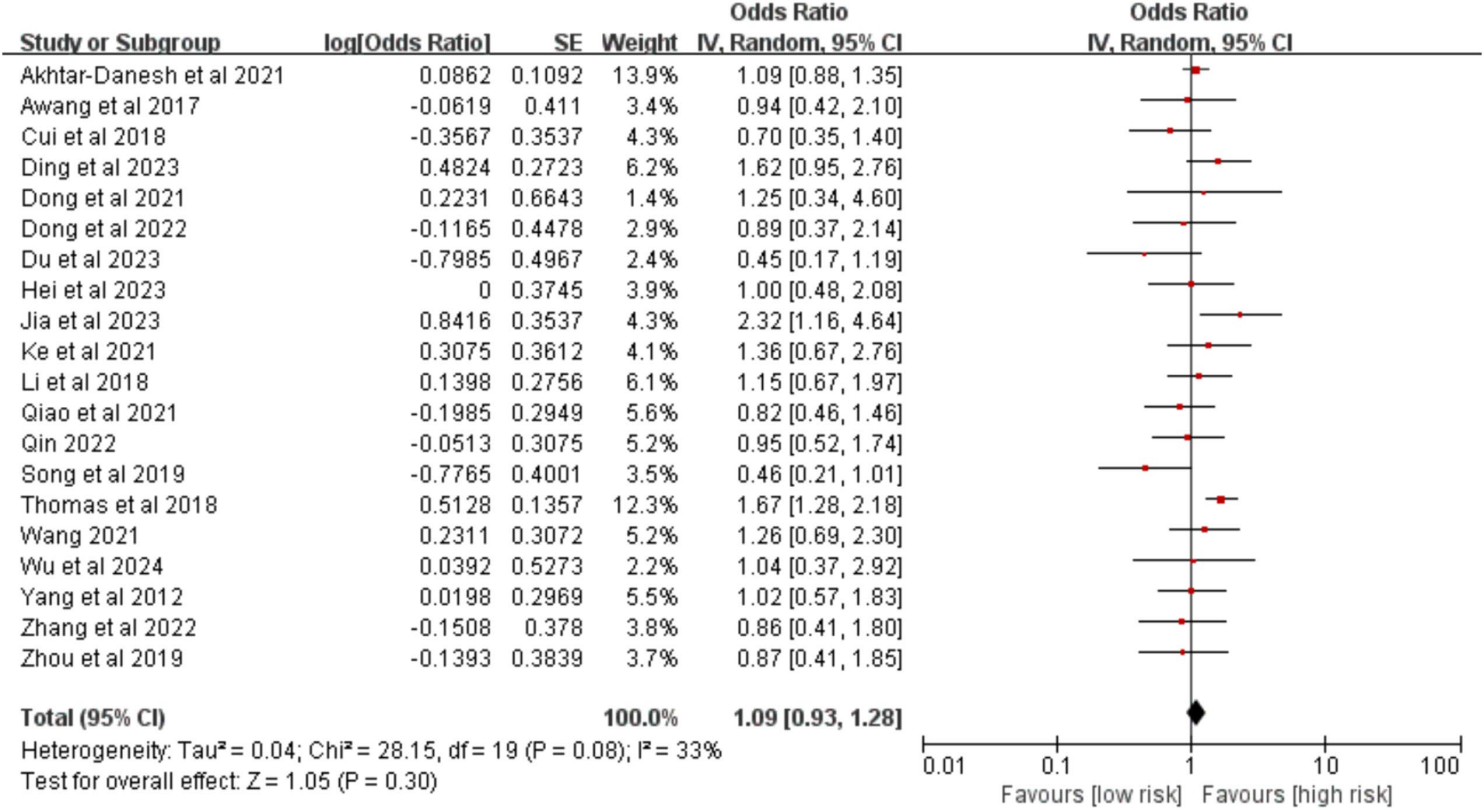
Meta-analysis of the association between sex and postoperative venous thromboembolism in patients with lung cancer.

Twenty studies were included in the analysis. The meta-analysis indicated that sex was not significantly associated with the risk of VTE following lung cancer surgery (OR = 1.09, 95% CI (0.93, 1.29), *p* = 0.300; Figure 2).

##### 3.2.1.2 Age

**Figure 3:**
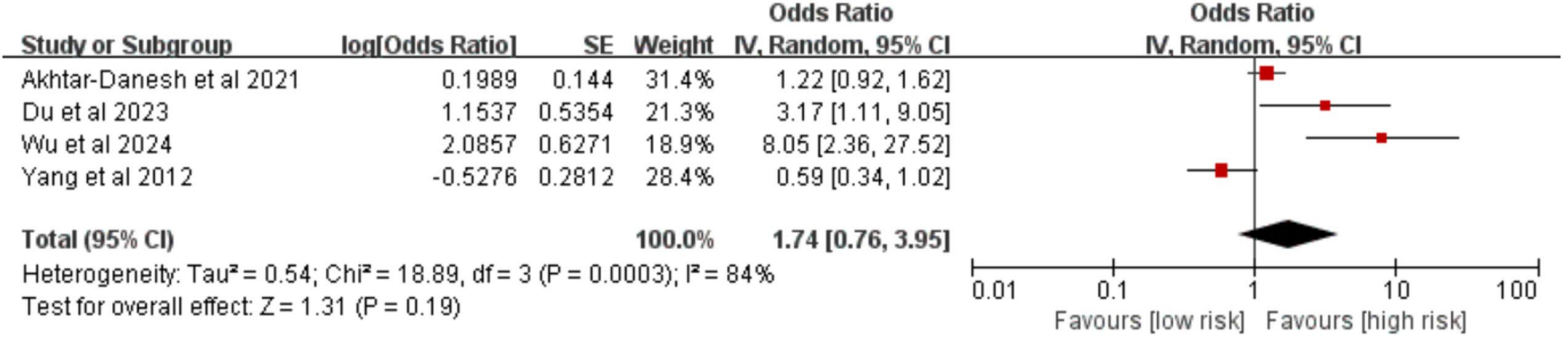
Meta-analysis of the association between age ≥ 60 years old and postoperative venous thromboembolism in patients with lung cancer.

**Figure 4:**
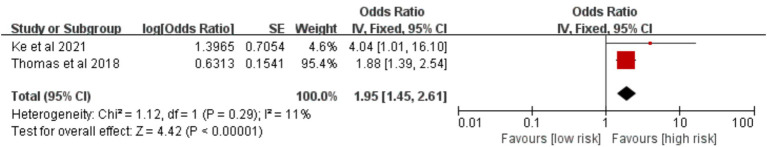
Meta-analysis of the association between age ≥ 65 years old and postoperative venous thromboembolism in patients with lung cancer.

A pooled analysis of six studies was performed to assess the influence of age. Based on four studies utilizing a threshold of 60 years, no significant association was found between age ≥ 60 years and postoperative VTE risk [OR = 1.74, 95% CI (0.76, 3.95), *p* = 0.190] (Figure 3). However, analysis of two studies that defined older age as ≥ 65 years identified it as a significant risk factor for VTE [OR = 1.95, 95% CI (1.45, 2.61), *p* < 0.00001] (Figure 4).

##### 3.2.1.3 History of smoking

**Figure 5:**
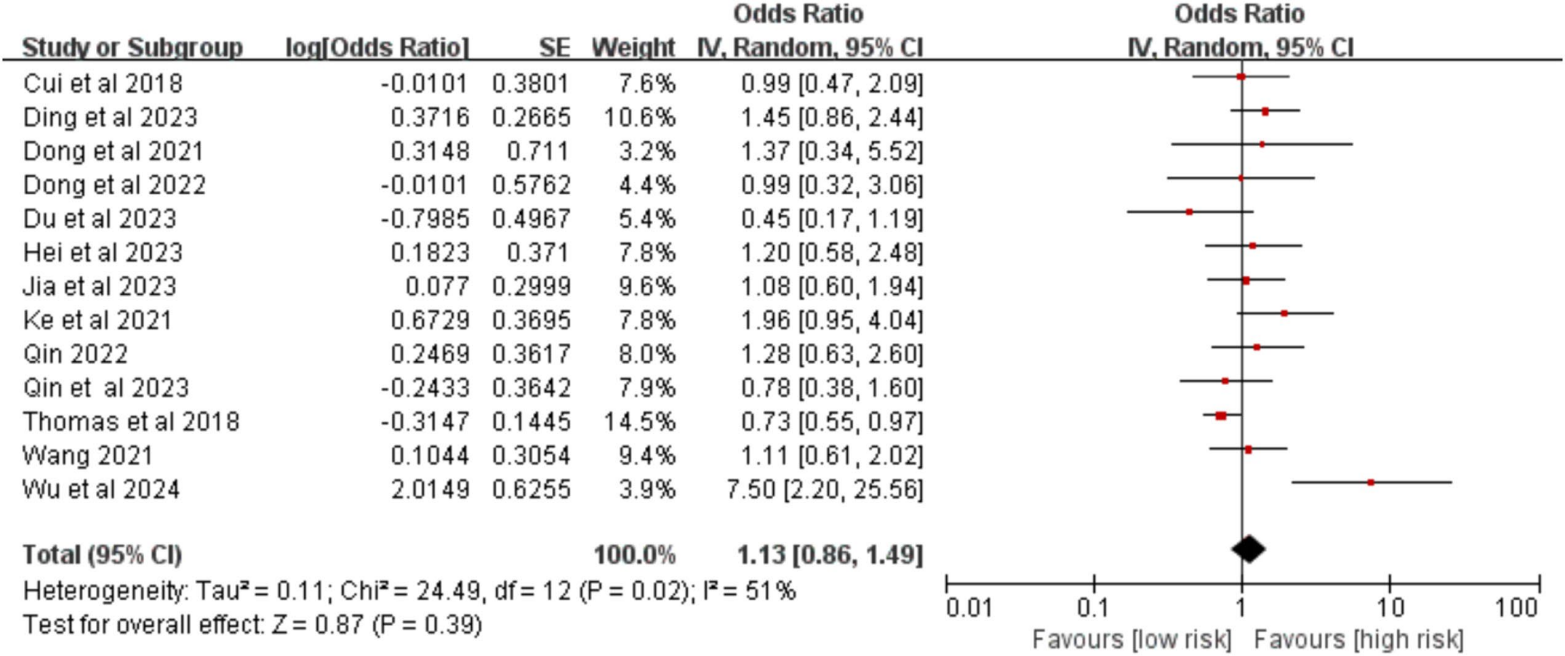
Meta-analysis of the association between smoking history and postoperative venous thromboembolism in patients with lung cancer.

Thirteen studies provided data on smoking history. The meta-analysis revealed no statistically significant association between a history of smoking and the development of VTE after surgery [OR = 1.13, 95% CI (0.86, 1.49), *p* = 0.390] (Figure 5).

##### 3.2.1.4 Drinking history

**Figure 6:**
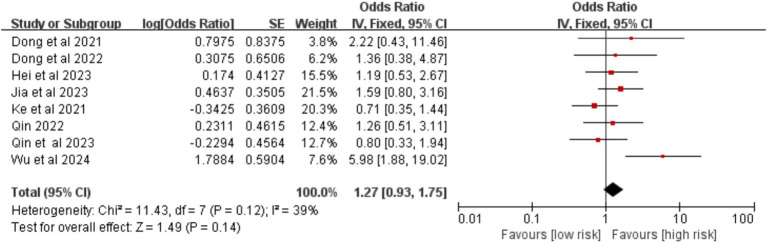
Meta-analysis of the association between drinking history and postoperative venous thromboembolism in patients with lung cancer.

Data from eight studies were analyzed for drinking history. The results showed no significant association between a history of drinking and postoperative VTE risk [OR = 1.27, 95% CI (0.93, 1.75), *p* = 0.140] (Figure 6).

##### 3.2.1.5 Body mass index (BMI)

**Figure 7:**
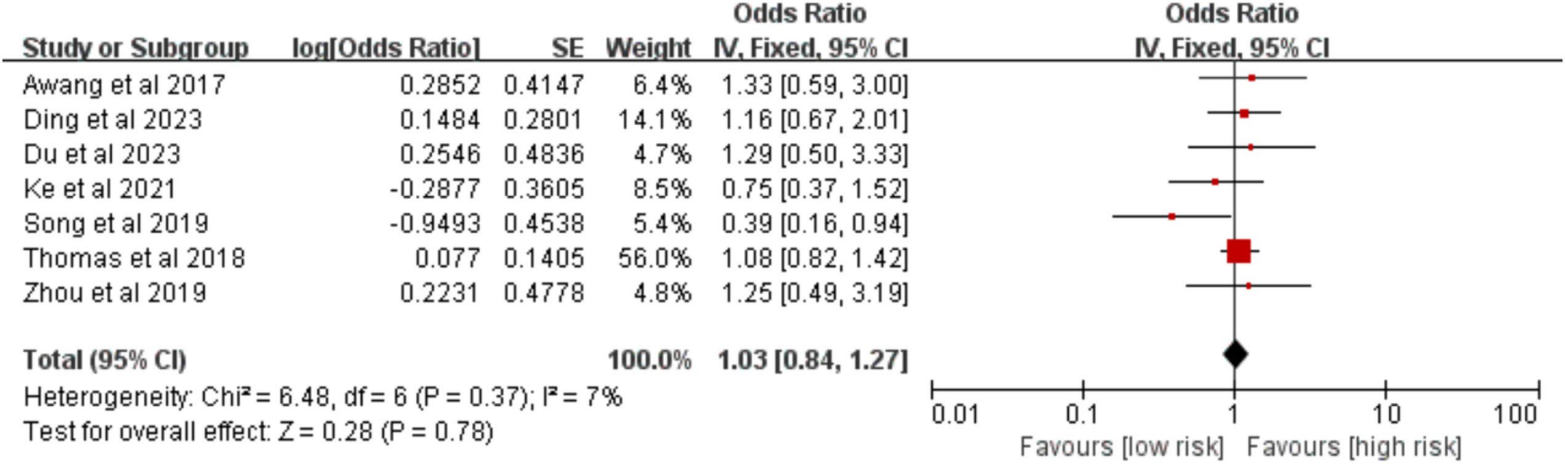
Meta-analysis of the association between body mass index ≥ 25 kg/m^2^ and postoperative venous thromboembolism in patients with lung cancer.

Seven studies were included to evaluate BMI. The meta-analysis demonstrated that a BMI ≥ 25 kg/m^2^ was not significantly associated with an increased risk of VTE [OR = 1.03, 95% CI (0.84, 1.27), *p* = 0.780] (Figure 7).

#### 3.2.2 Disease condition

##### 3.2.2.1 Hypertension

**Figure 8:**
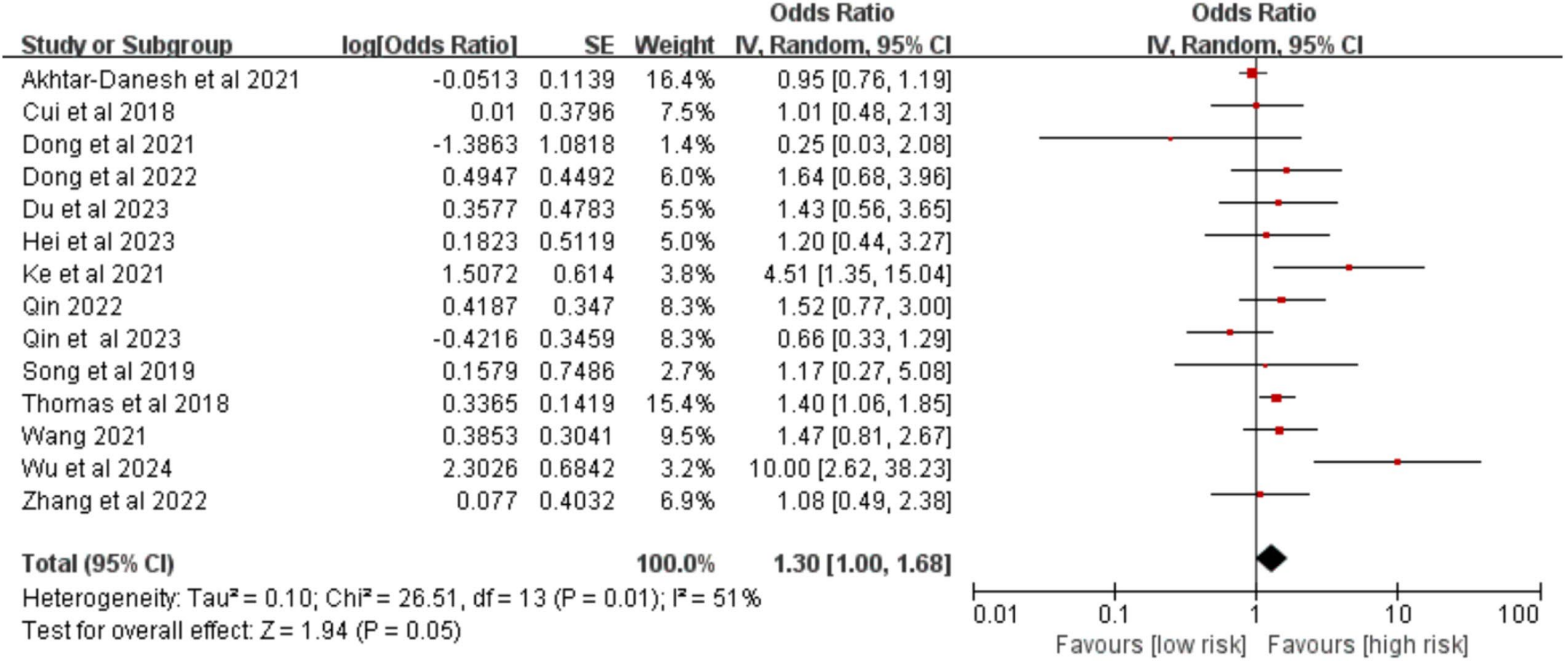
Meta-analysis of the association between hypertension and postoperative venous thromboembolism in patients with lung cancer.

Fourteen studies were included. The meta-analysis found no statistically significant association between hypertension and the risk of VTE after lung cancer surgery [OR = 1.30,95% CI (1.00,1.68), *p* = 0.050] (Figure 8).

##### 3.2.2.2 Hyperlipidemia

**Figure 9:**
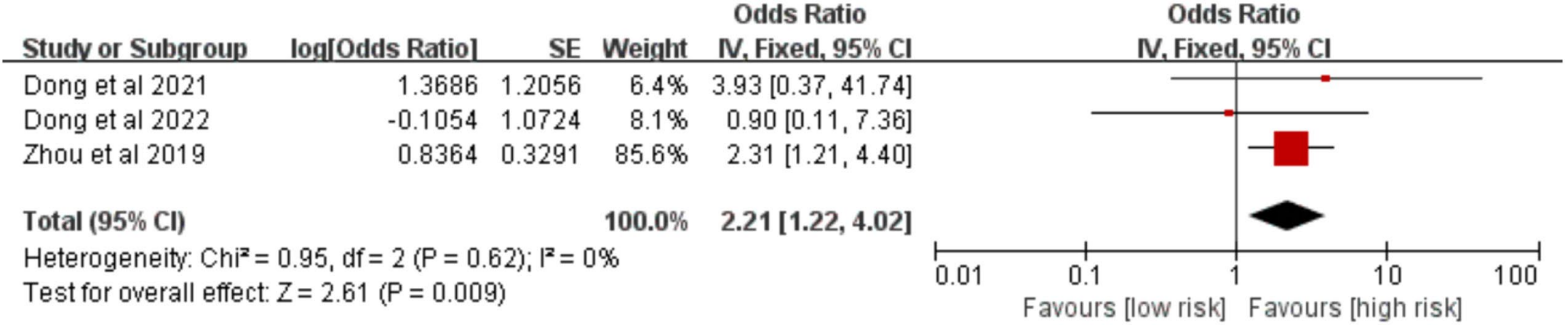
Meta-analysis of the association between hyperlipidemia and postoperative venous thromboembolism in patients with lung cancer.

Pooled results from three studies indicated that hyperlipidemia was a significant risk factor for VTE [OR = 2.21, 95% CI (1.22, 4.02), *p* = 0.009] (Figure 9).

##### 3.2.2.3 Tumor staging

**Figure 10:**
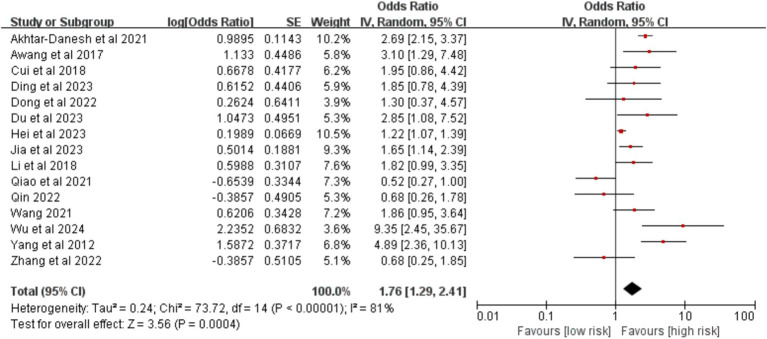
Meta-analysis of the association between tumor staging and postoperative venous thromboembolism in patients with lung cancer.

Analysis of 15 studies demonstrated that advanced tumor stage (III–IV) was significantly associated with an increased risk of VTE [OR = 1.76, 95%CI (1.29, 2.41), *p* = 0.0004] (Figure 10).

##### 3.2.2.4 Coronary heart disease

**Figure 11:**
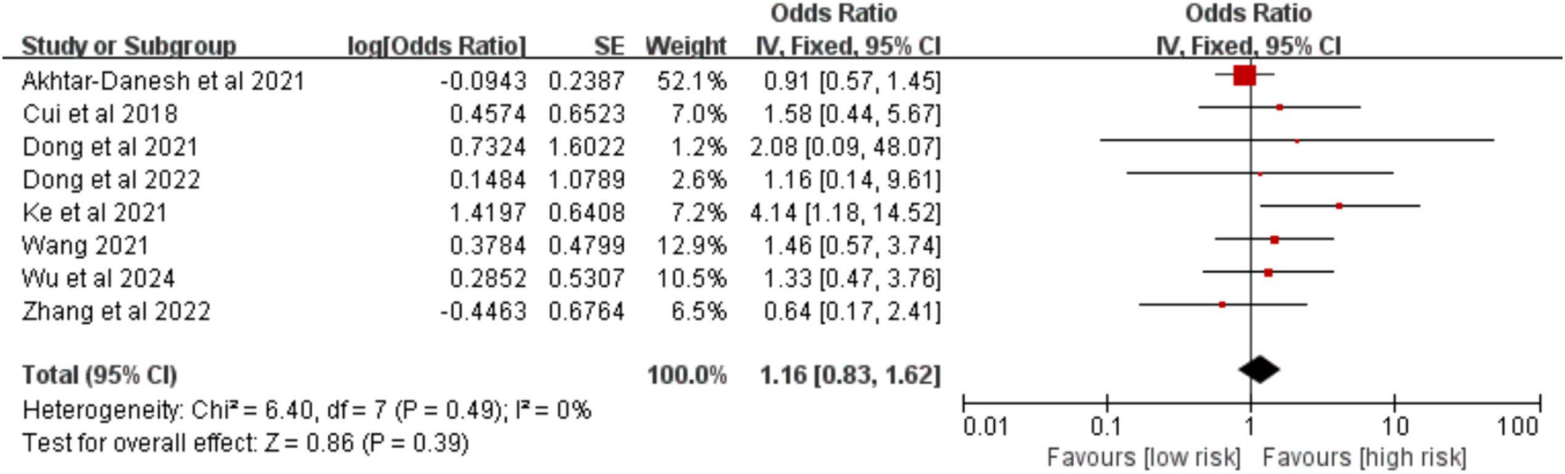
Meta-analysis of the association between coronary heart disease and postoperative venous thromboembolism in patients with lung cancer.

Data from eight studies were analyzed. The meta-analysis showed that coronary heart disease was not significantly associated with VTE risk [OR = 1.16, 95%CI (0.83, 1.62), *p* = 0.390] (Figure 11).

##### 3.2.2.5 Diabetes

**Figure 12:**
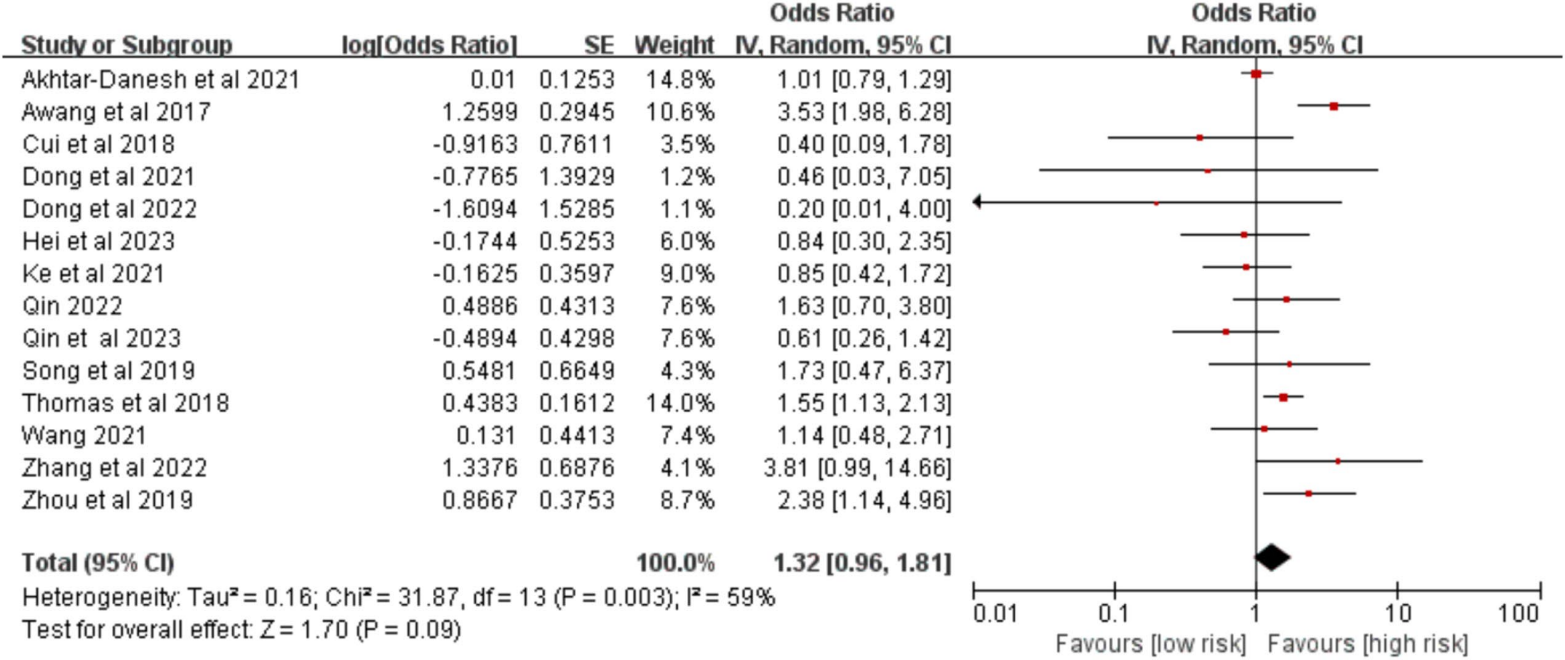
Meta-analysis of the association between diabetes and postoperative venous thromboembolism in patients with lung cancer.

Fourteen studies provided data on diabetes. No significant association was found between diabetes and postoperative VTE [OR = 1.32, 95%CI (0.96, 1.81), *p* = 0.090] (Figure 12).

##### 3.2.2.6 Pathological type

**Figure 13:**
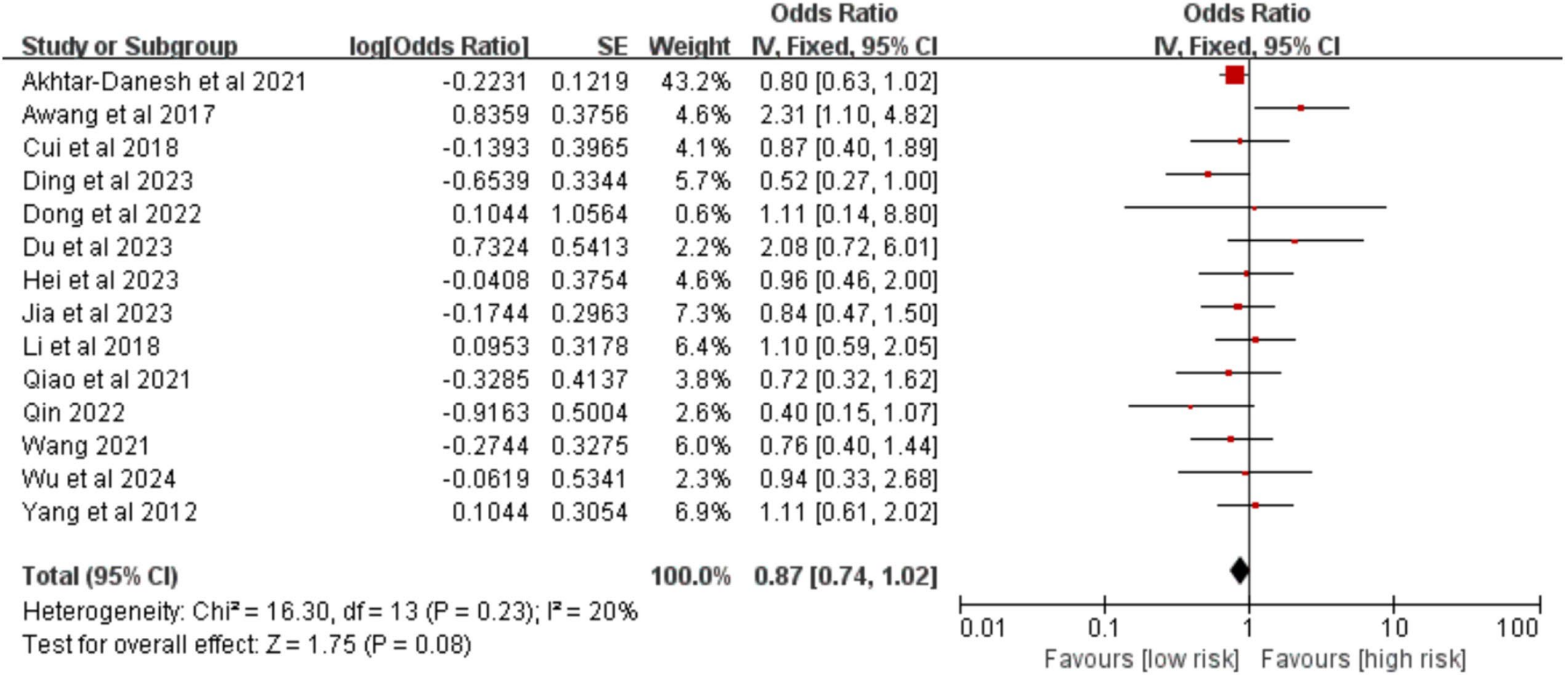
Meta-analysis of the association between pathological type and postoperative venous thromboembolism in patients with lung cancer.

Based on 14 studies, the pathological type (adenocarcinoma versus other types) was not significantly associated with VTE risk [OR = 0.87, 95%CI (0.74, 1.02), *p* = 0.080] (Figure 13).

#### 3.2.3 Surgery-related factors

##### 3.2.3.1 Type of surgery

**Figure 14:**
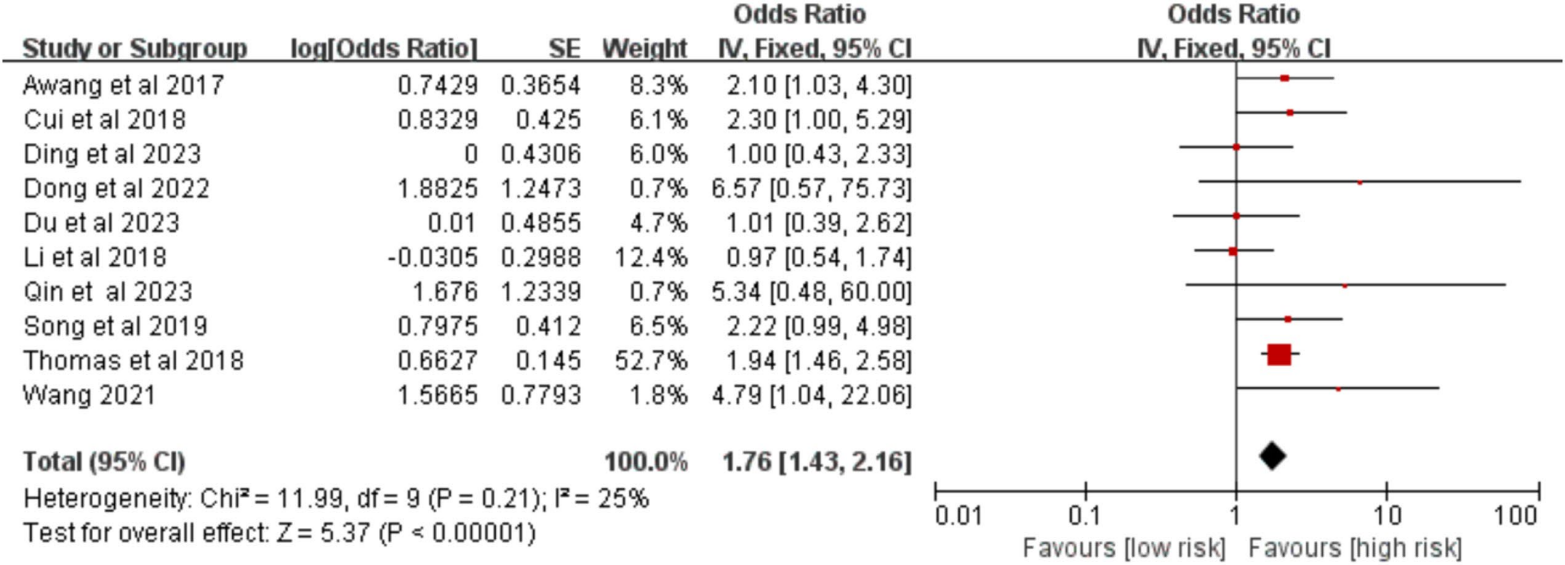
Meta-analysis of the association between the surgical approach and postoperative venous thromboembolism in patients with lung cancer.

Ten studies compared surgical approaches. The meta-analysis identified thoracotomy as a significant risk factor for VTE [OR = 1.76, 95% CI (1.43, 2.16), *p* < 0.00001] (Figure 14).

##### 3.2.3.2 Operation time

**Figure 15:**
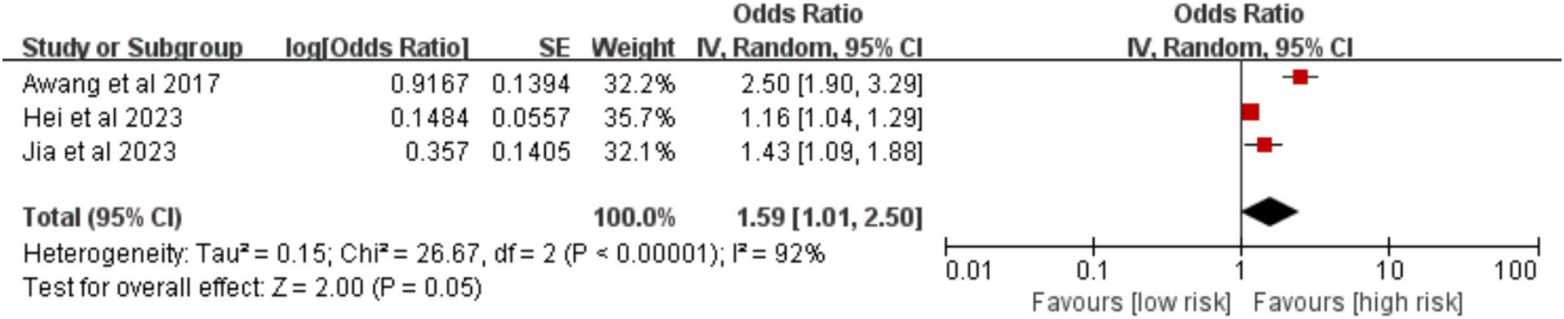
Meta-analysis of the association between operation time ≥ 3 h and postoperative venous thromboembolism in patients with lung cancer.

**Figure 16:**
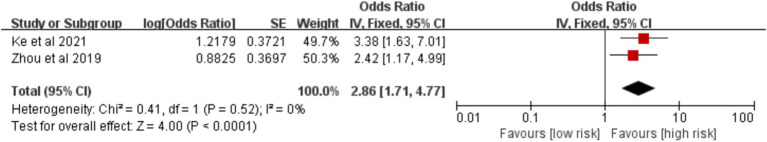
Meta-analysis of the association between operation time ≥ 2 h and postoperative venous thromboembolism in patients with lung cancer.

Five studies examined the effect of operation time. Analysis of three studies using a 3-h threshold showed no statistically significant association with VTE risk [OR = 1.59, 95%CI (1.01, 2.50), *p* = 0.050] (Figure 15). However, based on two studies using a 2-h threshold, an operation time ≥ 2 h was a significant risk factor [OR = 2.86, 95%CI (1.71, 4.77), *p* < 0.0001] (Figure 16).

##### 3.2.3.3 Intraoperative blood loss

Two studies reported intraoperative blood loss. The meta-analysis indicated that blood loss ≥ 200 mL was a significant risk factor for VTE.

**Figure 17:**
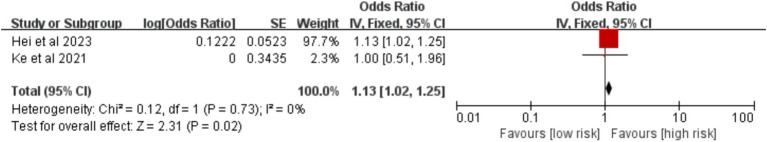
Meta-analysis of the association between intraoperative bleeding and postoperative venous thromboembolism in patients with lung cancer.

[OR = 1.13, 95%CI (1.02, 1.25), *p* = 0.020] (Figure 17).

##### 3.2.3.4 Tumor site

**Figure 18:**
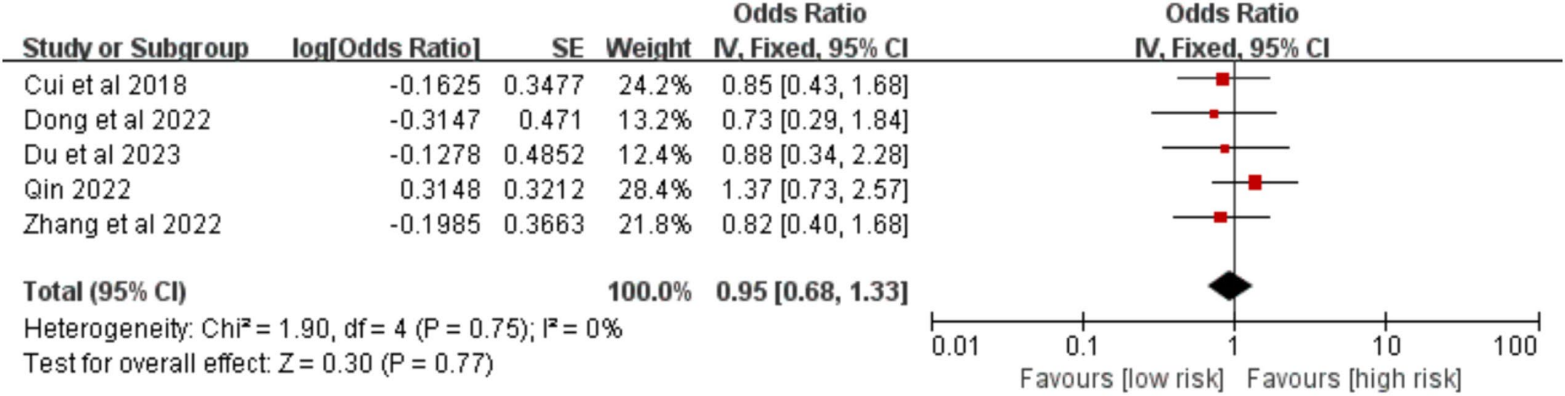
Meta-analysis of the association between tumor site and postoperative venous thromboembolism in patients with lung cancer.

Data from five studies showed that tumor location (left versus right lung) was not significantly associated with VTE risk [OR = 0.95, 95%CI (0.68, 1.33), *p* = 0.770] (Figure 18).

##### 3.2.3.5 Type of lung resection

**Figure 19:**
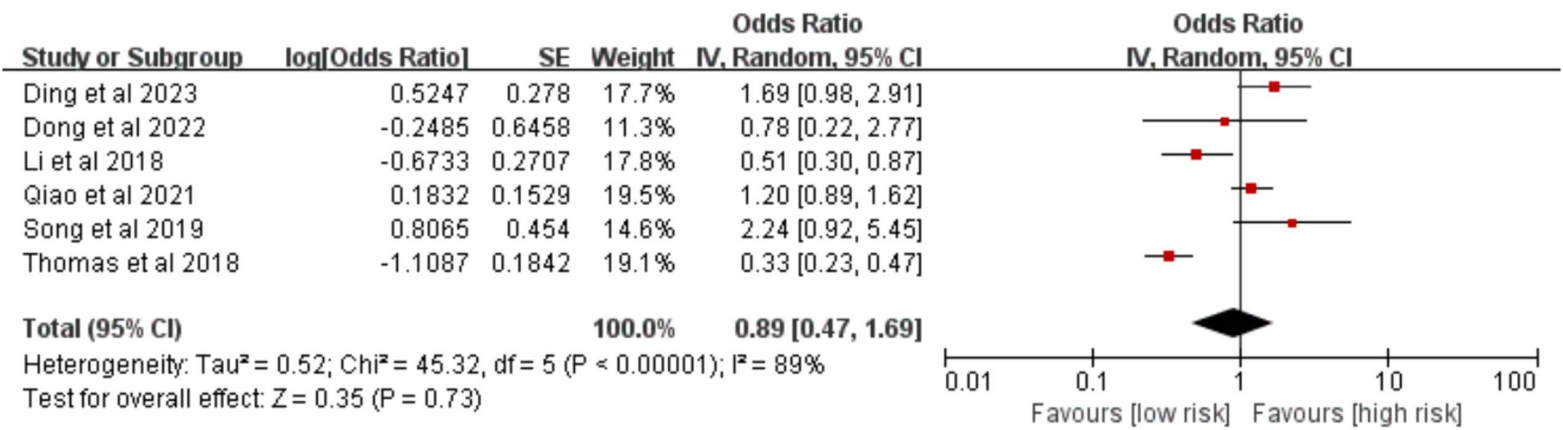
Meta-analysis of the association between type of lung resection and postoperative venous thromboembolism in patients with lung cancer.

Six studies were included. The meta-analysis found that the type of lung resection (lobectomy versus other resections) was not significantly associated with VTE risk [OR = 0.89, 95%CI (0.47, 1.69), *p* = 0.730] (Figure 19).

#### 3.2.4 Other factors

##### 3.2.4.1 Preoperative chemotherapy

**Figure 20:**
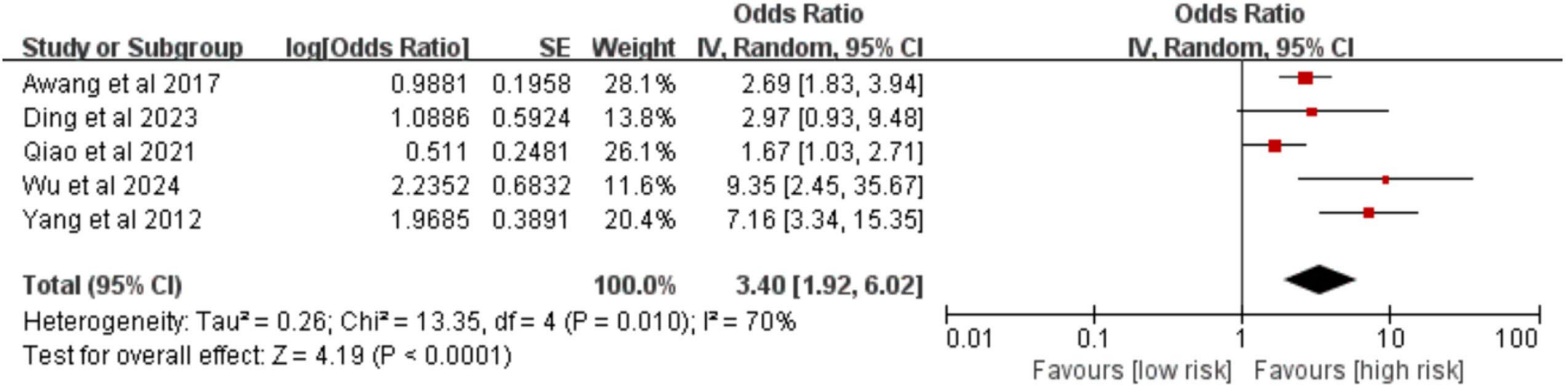
Meta-analysis of the association between preoperative chemotherapy and postoperative venous thromboembolism in patients with lung cancer.

Analysis of five studies showed that preoperative chemotherapy was a significant risk factor for VTE [OR = 3.40, 95%CI (1.92, 6.02), *p* < 0.0001] (Figure 20).

##### 3.2.4.2 Abnormal D-dimer

**Figure 21:**
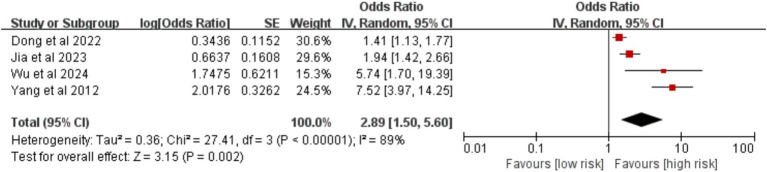
Meta-analysis of the association between D-dimer abnormality and postoperative venous thromboembolism in patients with lung cancer.

Based on four studies, an abnormal D-dimer level was identified as a significant risk factor for VTE [OR = 2.89, 95%CI (1.50, 5.60), *p* = 0.002] (Figure 21).

#### 3.2.5 Publication bias

**Figure 22:**
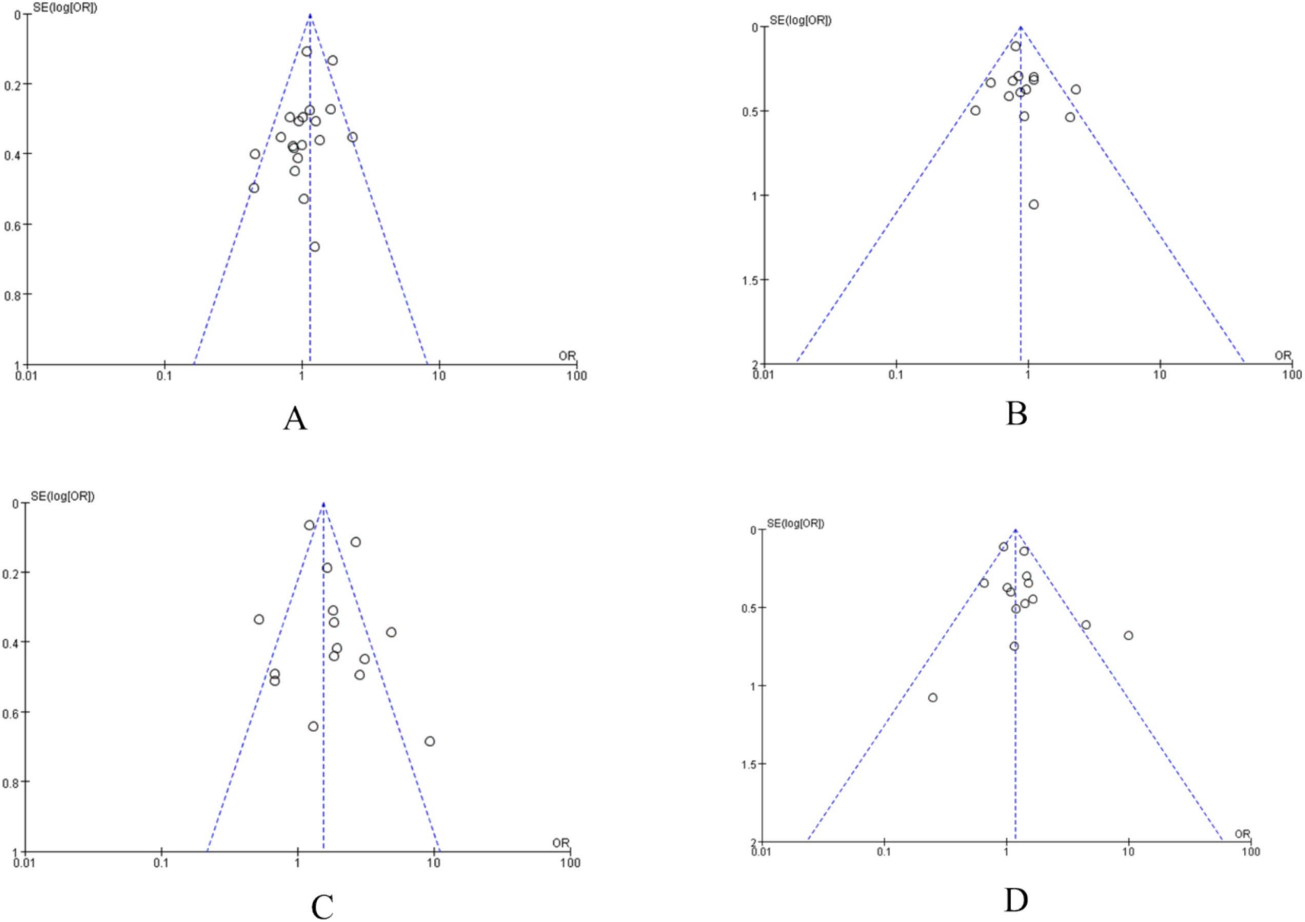
Funnel plot: **(A)** Sex; **(B)** pathological type; **(C)** tumor stage; and **(D)** hypertension.

Funnel plots were generated for factors with sufficient included studies, such as sex, pathological type, tumor stage, and hypertension. The scatter points in these funnel plots showed approximate symmetry, suggesting a low likelihood of significant publication bias (Figure 22).

#### 3.2.6 Sensitivity analysis

Sensitivity analysis, performed by alternating between fixed-effect and random-effects models, demonstrated that the direction and significance of the pooled results for all risk factors remained consistent. This indicates that the findings are robust and not overly dependent on the choice of statistical model (Table 2).

**Table 2:** Each outcome changed the model sensitivity analysis results.

Outcomes	*I^2^*	*p*-value	Pooled analysis results			Change model analysis results		
			Model	OR (95% CI)	*p*-value	Model	OR (95% Cl)	*p*-value
Sex (Male)	33%	0.080	Random	1.09 (0.93, 1.28)	0.300	Fixed	**1.15 (1.02, 1.29)**	**0.020**
Age (≥ 60 years)	84%	0.0003	Random	1.74 (0.76, 3.95)	0.190	Fixed	1.20 (0.95, 1.53)	0.130
Age (≥ 65 years)	11%	0.290	Fixed	**1.95 (1.45, 2.61)**	**<0.00001**	Random	**2.02 (1.30, 3.14)**	**0.002**
Smoking history	51%	0.020	Random	1.13 (0.86, 1.49)	0.390	Fixed	1.01 (0.85, 1.19)	0.940
Drinking history	39%	0.120	Fixed	1.27 (0.93, 1.75)	0.140	Random	1.34 (0.88, 2.04)	0.180
BMI (≥ 25 kg/m^2^)	7%	0.370	Fixed	1.03 (0.84, 1.27)	0.780	Random	1.02 (0.81, 1.28)	0.860
Hypertension history	51%	0.010	Random	1.30 (1.00, 1.68)	0.050	Fixed	**1.17 (1.02, 1.35)**	**0.030**
Hyperlipidemia	0	0.620	Fixed	**2.21 (1.22, 4.02)**	**0.009**	Random	**2.21 (1.22, 4.02)**	**0.009**
Tumor stage	81%	<0.00001	Random	**1.76 (1.29, 2.41)**	**0.0004**	Fixed	**1.54 (1.39, 1.70)**	**<0.00001**
Coronary heart disease	0	0.490	Fixed	1.16 (0.83, 1.62)	0.390	Random	1.16 (0.83, 1.62)	0.390
Diabetes	59%	0.003	Random	1.32 (0.96, 1.81)	0.090	Fixed	**1.28 (1.09, 1.50)**	**0.002**
Adenocarcinoma	20%	0.230	Fixed	0.87 (0.74, 1.02)	0.080	Random	0.89 (0.73, 1.09)	0.270
Thoracotomy	25%	0.210	Fixed	**1.76 (1.43, 2.16)**	**<0.00001**	Random	**1.72 (1.29, 2.29)**	**0.0002**
Operative time (≥ 3 h)	92%	<0.00001	Random	1.59 (1.01, 2.50)	0.050	Fixed	**1.31 (1.19, 1.44)**	**<0.00001**
Operative time (≥ 2 h)	0	0.520	Fixed	**2.86 (1.71, 4.77)**	**<0.0001**	Random	**2.86 (1.71, 4.77)**	**<0.0001**
Intraoperative bleeding (≥ 200 ml)	0	0.730	Fixed	**1.13 (1.02, 1.25)**	**0.020**	Random	**1.13 (1.02, 1.25)**	**0.020**
Tumor site(Left lung)	0	0.750	Fixed	0.95 (0.68, 1.33)	0.770	Random	0.95 (0.68, 1.33)	0.770
Pulmonary lobe resection	89%	<0.00001	Random	0.89 (0.47, 1.69)	0.730	Fixed	**0.80 (0.66, 0.97)**	**0.020**
History of chemotherapy	70%	0.010	Random	**3.40 (1.92, 6.02)**	**<0.0001**	Fixed	**2.77 (2.12, 3.62)**	**<0.00001**
D-dimer abnormality	89%	<0.00001	Random	**2.89 (1.50, 5.60)**	**0.002**	Fixed	**1.81 (1.52, 2.16)**	**<0.00001**

OR, Odds ratio; CL, Confidence interval; BMI, Body mass index. Bolded odds ratios (OR) indicate a statistically significant association under the specific model (fixed- or random-effects) applied for that risk factor in the analysis.

## 4 Discussion

This meta-analysis, encompassing 21 studies with a total of 41,780 participants, evaluated 18 potential risk factors for VTE following lung cancer surgery. The results identified the following significant risk factors: age ≥ 65 years, hyperlipidemia, tumor stage III–IV, thoracotomy, operation time ≥ 2 h, intraoperative blood loss ≥ 200 mL, preoperative chemotherapy, and abnormal D-dimer levels.

Previous studies have consistently recognized advanced age as a risk factor for postoperative VTE in lung cancer patients (13, 16, 25, 31). However, the specific age threshold for risk stratification remains controversial. Our analysis demonstrated that patients aged 65 years or older had a significantly higher risk of VTE. This may be explained by age-related physiological decline, including diminished functional reserve, reduced muscle tone, endothelial dysfunction, and impaired venous compliance, all of which contribute to an elevated thromboembolic risk in the elderly surgical population (35). While some studies (13) have suggested smoking as a risk factor due to its role in vascular endothelial injury, platelet activation, increased blood viscosity, and slowed blood flow, thereby accelerating thrombosis (36), our meta-analysis did not find a statistically significant association between smoking history and VTE. We believe that the studies included might have only regarded smoking history as a binary variable of “present/absent,” failing to incorporate more precise indicators of exposure dose. Moreover, in the specific group of lung cancer patients, smoking itself is the primary causative factor (37). The baseline smoking rate among the study population was generally high, which might have weakened the effectiveness of the comparison between the groups. Similarly, although male sex has been linked to higher VTE incidence—possibly due to a higher prevalence of smoking and associated increases in blood viscosity with the long-term effect of nicotine in tobacco (21)—our results did not identify male sex as an independent risk factor.

Hyperlipidemia was confirmed as a significant risk factor for VTE after lung cancer surgery in this study. The underlying mechanism may involve vascular endothelial injury and enhanced platelet aggregation caused by high lipid levels. Elevated cholesterol and triglycerides can contribute to atherosclerotic plaque formation, narrowing the vascular lumen, impeding blood flow, and limiting postoperative mobility, thereby increasing thrombotic risk (38). Furthermore, advanced tumor stage (III–IV) was strongly associated with VTE, consistent with earlier reports such as that by Amer et al., who observed VTE incidences of 64.8% in stage III–IV patients compared to 34.2% in stage I–II patients (39). This may be attributed to cancer progression and metastasis exacerbating systemic hypercoagulability (20). Although some evidence suggests that lung adenocarcinoma carries a higher VTE risk compared to squamous cell carcinoma (40), our analysis did not find a statistically significant association between its pathological subtype and postoperative VTE.

Our study identified several surgery-related factors as significant contributors to VTE risk. Specifically, thoracotomy, an operation time ≥ 2 h, and intraoperative blood loss ≥ 200 mL were all independently associated with an increased incidence of VTE following lung cancer surgery. During lung cancer surgery, the clamping and manipulation of major blood vessels can cause trauma to local arteries and veins. Compared to video-assisted thoracic surgery, open thoracotomy inevitably leads to more extensive tissue damage, which elevates systemic stress levels and promotes the release of inflammatory factors. This inflammatory response can induce endothelial cell dysfunction, thereby activating the coagulation system and ultimately promoting thrombus formation (41). Consequently, lung cancer patients undergoing thoracotomy are at a higher risk of developing VTE. Prolonged operative time directly extends both anesthesia duration and immobilization period, which may cause vascular endothelial injury and alter hemodynamics, leading to reduced venous pressure and decreased blood flow velocity, thereby increasing the risk of thrombosis (42, 43). However, the findings of this study indicated that while an operative time of ≥2 h was a risk factor for VTE, a threshold of ≥3 h did not show statistical significance. This discrepancy may be attributed to the limited number of studies included. Increased intraoperative blood loss leads to hemoconcentration, and concomitant peripheral vasoconstriction slows blood flow, collectively promoting thrombosis (44). Additionally, prolonged bed rest after surgery can cause reduced and stagnant blood flow in the lower extremity veins, which may also elevate the risk of thrombosis (45).

Studies have shown that there is a statistically significant positive correlation between BMI and VTE (46). However, in oncology, there is a notable “obesity paradox” phenomenon (47). For lung cancer patients, obesity is associated with better postoperative prognosis and lower incidence. Patients with mild overweight may have a survival advantage when dealing with surgical stress due to better metabolic reserves and nutritional status (47, 48).

Preoperative chemotherapy was also identified as a significant risk factor in our study. Platinum-based agents, in particular, are known to enhance thrombin generation and reduce levels of natural anticoagulants such as proteins S and C. Many chemotherapeutic drugs can directly injure vascular endothelial cells, activate the coagulation system, and suppress fibrinolysis, collectively increasing thrombosis risk (49, 50). Finally, elevated D-dimer—a fibrin degradation product reflecting fibrinolytic activity—was confirmed as a risk factor in this study. It serves as an important biomarker in the diagnosis of thrombosis and pulmonary microvascular embolism (51), and our results support its value in predicting VTE after lung cancer surgery.

## 5 Limitations

This study has several limitations (1): Only Chinese and English publications were included, potentially overlooking relevant studies in other languages and introducing selection bias (2). Some risk factors (e.g., age ≥ 65 years old, operation time ≥ 2 h, operation time ≥ 3 h, hyperlipidemia, and intraoperative blood loss ≥200 mL) were analyzed based on a limited number of studies and small sample sizes. Due to the limited number of included studies, this may affect the stability of the combined effect values and statistical power. Therefore, the interpretation of the strength of association for these specific factors should be cautious (3). Variability in follow-up durations across the included studies may have affected the accuracy of postoperative VTE incidence estimates (4). Due to the limited availability of data, it was not feasible to evaluate all potential risk factors (5). Specific genetic mutations (e.g., ALK, EGFR, and KRAS) and related targeted therapies (such as EGFR-TKI inhibitors) may have distinct associations with thrombotic risk; however, due to insufficient reporting in the original studies, this aspect could not be incorporated into our analysis. Therefore, it is suggested that multi-center and large-sample epidemiological studies be carried out in the future to further clarify the related risk factors for VTE in patients after lung cancer surgery.

## 6 Conclusion

This meta-analysis identified the following significant risk factors for postoperative VTE in lung cancer patients: age ≥ 65 years, hyperlipidemia, advanced tumor stage (III–IV), thoracotomy, operation time ≥ 2 h, intraoperative blood loss ≥ 200 mL, abnormal D-dimer levels, and preoperative chemotherapy. Prior to scheduling surgery, clinicians should thoroughly evaluate whether patients exhibit these risk factors to mitigate the incidence of VTE. Future multi-center, large-sample epidemiological studies are recommended to further elucidate the risk factors associated with VTE in this patient population.

## Data Availability

The original contributions presented in the study are included in the article/Supplementary material, further inquiries can be directed to the corresponding author/s.
