# Supplementary material for: Risk factors for postoperative venous thromboembolism in patients with lung cancer: a systematic review and meta-analysis
Source: Front Med (Lausanne). 2026 Jan 14;12:1699892. doi: 10.3389/fmed.2025.1699892 (PMC12846959; doi:10.3389/fmed.2025.1699892)
Supplement: Supplementary file 1 [file Supplementary_file_1.pdf]

Supplementary material:

Table S1 Search strategy in PubMed.

Search	Query
#1	Lung Neoplasms[MeSH Terms] OR Lung Neoplasms[Title/Abstract] OR Neoplasms, Pulmonary[Title/Abstract] OR Neoplasm, Pulmonary[Title/Abstract] OR Pulmonary Neoplasm[Title/Abstract] OR Pulmonary Neoplasms[Title/Abstract] OR Neoplasms, Lung[Title/Abstract] OR Lung Neoplasm[Title/Abstract] OR Neoplasm, Lung[Title/Abstract] OR Lung Cancer [Title/Abstract] OR Cancer, Lung[Title/Abstract] OR Cancers, Lung[Title/Abstract] OR Lung Cancers[Title/Abstract] OR Pulmonary Cancer[Title/Abstract] OR Cancer, Pulmonary[Title/Abstract] OR Cancers, Pulmonary[Title/Abstract] OR Pulmonary Cancers[Title/Abstract]
#2	Pulmonary Embolism[MeSH Terms] OR Pulmonary Embolism[Title/Abstract] OR Embolism, Pulmonary[Title/Abstract] OR Embolisms, Pulmonary[Title/Abstract] OR Pulmonary Embolisms[Title/Abstract] OR Pulmonary Thromboembolisms[Title/Abstract] OR Thromboembolisms, Pulmonary[Title/Abstract] OR Pulmonary Thromboembolism[Title/Abstract] OR Thromboembolism, Pulmonary[Title/Abstract] OR Venous Thrombosis[MeSH Terms] OR Venous Thrombosis[Title/Abstract] OR Thrombosis, Venous[Title/Abstract] OR Thromboses, Venous[Title/Abstract] OR Venous Thromboses[Title/Abstract] OR Phlebothrombosis[Title/Abstract] OR Phlebothromboses[Title/Abstract] OR Deep Vein Thrombosis[Title/Abstract] OR Deep Vein Thromboses[Title/Abstract] OR Thromboses, Deep Vein[Title/Abstract] OR Vein Thromboses, Deep[Title/Abstract] OR Vein Thrombosis, Deep[Title/Abstract] OR Thrombosis, Deep Vein[Title/Abstract] OR Deep Venous Thrombosis[Title/Abstract] OR Deep Venous Thromboses[Title/Abstract] OR Thromboses, Deep Venous[Title/Abstract] OR Thrombosis, Deep Venous[Title/Abstract] OR Venous Thromboses, Deep[Title/Abstract] OR Venous Thrombosis, Deep[Title/Abstract] OR Deep-Vein Thrombosis[Title/Abstract] OR Deep-Vein Thromboses[Title/Abstract] OR Thrombosis, Deep-Vein[Title/Abstract] OR Deep-Venous Thrombosis[Title/Abstract] OR Deep-Venous Thromboses[Title/Abstract] OR Thromboses, Deep-Venous[Title/Abstract] OR Thrombosis, Deep-Venous[Title/Abstract] OR Venous Thromboembolism[MeSH Terms] OR Thromboembolism, Venous[Title/Abstract] OR Venous Thromboembolism[Title/Abstract]
#3	Risk Factors[MeSH Terms] OR Factor, Risk[Title/Abstract] OR Risk Factors[Title/Abstract] OR Risk Factor[Title/Abstract] OR Risk Scores[Title/Abstract] OR Risk Score[Title/Abstract] OR Score, Risk[Title/Abstract] OR Risk Factor Scores[Title/Abstract] OR Risk Factor Score[Title/Abstract] OR Score, Risk Factor[Title/Abstract] OR Health Correlates[Title/Abstract] OR Correlates, Health[Title/Abstract] OR Social Risk Factors[Title/Abstract] OR Factor, Social Risk[Title/Abstract] OR Factors, Social Risk[Title/Abstract] OR Risk Factor, Social[Title/Abstract] OR Risk Factors, Social[Title/Abstract] OR Social Risk Factor[Title/Abstract]
#4	#1 AND #2 AND #3

Table S2 Search strategy in Embase.

Search	Query
#1	'lung cancer'/exp OR 'broncho-pulmonary cancer':ab,ti OR 'bronchopulmonary cancer':ab,ti OR 'ca lung':ab,ti OR 'cancer of the lung':ab,ti OR 'cancer, lung':ab,ti OR 'carcinogenesis of the lung':ab,ti OR 'lung malignancies':ab,ti OR 'lung malignancy':ab,ti OR 'malignancies of the lung':ab,ti OR 'malignancy of the lung':ab,ti OR 'malignant lung neoplasm':ab,ti OR 'malignant lung tumor':ab,ti OR 'malignant neoplasm of the lung':ab,ti OR 'malignant tumor of the lung':ab,ti OR 'pulmonary cancer':ab,ti OR 'pulmonary malignancies':ab,ti OR 'pulmonary malignancy':ab,ti OR 'schneeberg disease':ab,ti OR 'schneeberg lung disease':ab,ti OR 'lung cancer':ab,ti
#2	'venous thromboembolism'/exp OR 'thromboembolism, venous':ab,ti OR 'vein thromboembolism':ab,ti OR 'venous thromboembolism':ab,ti OR 'deep vein thrombosis'/exp OR 'acute deep venous thrombosis':ab,ti OR 'acute dvt':ab,ti OR 'deep thrombo-phlebitis':ab,ti OR 'deep thrombophlebitis':ab,ti OR 'deep vein blood clots':ab,ti OR 'deep vein thrombophlebitis':ab,ti OR 'deep vein thrombus':ab,ti OR 'deep venous thrombophlebitis':ab,ti OR 'deep venous thrombosis':ab,ti OR 'deep venous thrombus':ab,ti OR dvt:ab,ti OR 'deep vein thrombosis':ab,ti OR 'recurrent dvt':ab,ti OR 'thrombosis, acute deep venous':ab,ti OR 'deep vein thrombosis':ab,ti OR 'lung embolism'/exp OR 'chronic lung embolism':ab,ti OR 'embolism, lung':ab,ti OR 'lung embolization':ab,ti OR 'lung embolus':ab,ti OR 'lung embolus recurrence':ab,ti OR 'lung emboly':ab,ti OR 'lung microembolism':ab,ti OR 'lung microembolization':ab,ti OR 'lung microembolus':ab,ti OR 'lung thromboembolism':ab,ti OR 'microembolus, lung':ab,ti OR 'pulmonary embolism':ab,ti OR 'pulmonary embolization':ab,ti OR 'pulmonary embolus':ab,ti OR 'pulmonary microembolism':ab,ti OR 'pulmonary thromboembolic disease':ab,ti OR 'pulmonary thromboembolism':ab,ti OR 'thromboembolism, lung':ab,ti OR 'lung embolism':ab,ti
#3	'risk factor'/exp OR 'relative risk':ab,ti OR 'risk factors':ab,ti OR 'risk factor':ab,ti
#4	#1 AND #2 AND #3

Table S3 Search strategy in The Cochrane Library.

Search	Query
#1	MeSH descriptor: [Lung Neoplasms] explode all trees
#2	(Neoplasm, Pulmonary):ti,ab,kw OR (Lung Neoplasm):ti,ab,kw OR (Pulmonary Neoplasms):ti,ab,kw OR (Neoplasms, Lung):ti,ab,kw OR (Neoplasm, Lung):ti,ab,kw OR (Pulmonary Neoplasm):ti,ab,kw OR (Neoplasms, Pulmonary):ti,ab,kw OR (Lung Cancers):ti,ab,kw OR (Cancer of the Lung):ti,ab,kw OR (Pulmonary Cancers):ti,ab,kw OR (Lung Cancer):ti,ab,kw OR (Cancers, Lung):ti,ab,kw OR (Cancer, Lung):ti,ab,kw OR (Cancer of Lung):ti,ab,kw OR (Cancers, Pulmonary):ti,ab,kw OR (Cancer, Pulmonary):ti,ab,kw OR (Pulmonary Cancer):ti,ab,kw
#3	#1 OR #2
#4	MeSH descriptor: [Venous Thromboembolism] explode all trees
#5	(Thromboembolism, Venous):ti,ab,kw
#6	#4 OR #5
#7	MeSH descriptor: [Venous Thrombosis] explode all trees
#8	(Venous Thrombosis, Deep):ti,ab,kw OR (Thrombosis, Deep Venous):ti,ab,kw OR (Deep-Vein Thrombosis):ti,ab,kw OR (Deep-Venous Thrombosis):ti,ab,kw OR (Deep Venous Thromboses):ti,ab,kw OR (Thrombosis, Deep Vein):ti,ab,kw OR (Deep-Venous Thromboses):ti,ab,kw OR (Vein Thromboses, Deep):ti,ab,kw OR (Vein Thrombosis, Deep):ti,ab,kw OR (Thromboses, Deep-Venous):ti,ab,kw OR (Deep Vein Thrombosis):ti,ab,kw OR (Thrombosis, Deep-Venous):ti,ab,kw OR (Thromboses, Deep Vein):ti,ab,kw OR (Thromboses, Deep-Vein):ti,ab,kw OR (Thrombosis, Deep-Vein):ti,ab,kw OR (Thromboses, Deep Venous):ti,ab,kw OR (Venous Thromboses, Deep):ti,ab,kw OR (Deep-Vein Thromboses):ti,ab,kw OR (Deep Venous Thrombosis):ti,ab,kw OR (Deep Vein Thromboses):ti,ab,kw OR (Phlebothromboses):ti,ab,kw OR (Thrombosis, Venous):ti,ab,kw OR (Phlebothrombosis):ti,ab,kw OR (Venous Thromboses):ti,ab,kw OR (Thromboses, Venous):ti,ab,kw
#9	#7 OR #8
#10	MeSH descriptor: [Pulmonary Embolism] explode all trees
#11	(Pulmonary Thromboembolisms):ti,ab,kw OR (Thromboembolisms, Pulmonary):ti,ab,kw OR (Pulmonary Thromboembolism):ti,ab,kw OR (Thromboembolism, Pulmonary):ti,ab,kw OR (Embolism, Pulmonary):ti,ab,kw OR (Embolisms, Pulmonary):ti,ab,kw OR (Pulmonary Embolisms):ti,ab,kw
#12	#10 OR #11
#13	#6 OR #9 OR #12
#14	MeSH descriptor: [Risk Factors] explode all trees
#15	(Factor, Risk):ti,ab,kw OR (Risk Factor):ti,ab,kw OR (Correlates, Health):ti,ab,kw OR (Health Correlates):ti,ab,kw OR (Populations at Risk):ti,ab,kw OR (Population at Risk):ti,ab,kw OR (Risk Factors, Social):ti,ab,kw OR (Social Risk Factors):ti,ab,kw OR (Risk Factor, Social):ti,ab,kw OR (Factor, Social Risk):ti,ab,kw OR (Factors, Social Risk):ti,ab,kw OR (Social Risk Factor):ti,ab,kw OR

	(Risk Factor Scores):ti,ab,kw OR (Score, Risk Factor):ti,ab,kw OR (Risk Scores):ti,ab,kw OR (Risk Score):ti,ab,kw OR (Risk Factor Score):ti,ab,kw OR (Score, Risk):ti,ab,kw
#16	#14 OR #15
#17	#3 AND #13 AND #16

Table S4 Search strategy in Web of Science.

Search	Query
#1	TS=(Lung Neoplasms) OR TS=(Pulmonary Neoplasm) OR TS=(Pulmonary Neoplasms) OR TS=(Lung Neoplasm) OR TS=(Lung Cancer) OR TS=(Lung Cancers) OR TS=(Cancer of Lung) OR TS=(Pulmonary Cancer) OR TS=(Pulmonary Cancers) OR TS=(Cancer of the Lung)
#2	TS=(Venous Thromboembolism) OR TS=(Venous Thrombosis) OR TS=(Venous Thromboses) OR TS=(Phlebothrombosis) OR TS=(Phlebothromboses) OR TS=(Deep Vein Thrombosis) OR TS=(Deep Vein Thromboses) OR TS=(Deep Venous Thrombosis) OR TS=(Deep Venous Thromboses) OR TS=(Deep-Vein Thrombosis) OR TS=(Deep-Vein Thromboses) OR TS=(Deep-Venous Thrombosis) OR TS=(Deep-Venous Thromboses) OR TS=(Pulmonary Embolism) OR TS=(Pulmonary Embolisms) OR TS=(Pulmonary Thromboembolisms) OR TS=(Pulmonary Thromboembolism)
#3	TS=(Risk Factors) OR TS=(Risk Factor) OR TS=(Population at Risk) OR TS=(Populations at Risk) OR TS=(Risk Scores) OR TS=(Risk Score) OR TS=(Risk Factor Scores) OR TS=(Risk Factor Score) OR TS=(Health Correlates) OR TS=(Social Risk Factors) OR TS=(Social Risk Factor)
#4	#3 AND #2 AND #1

Table S5 Search strategy in CBM.

Search	Query
#1	("肺部癌症"[常用字段:智能] OR "肺癌"[常用字段:智能] OR "肺部肿瘤"[常用字段:智能] OR "肺肿瘤"[常用字段:智能])
#2	("静脉血栓栓塞"[常用字段:智能] OR "静脉血栓形成"[常用字段:智能]) OR ("深静脉血栓"[常用字段:智能] OR "肺栓塞"[常用字段:智能])
#3	("危险因素"[常用字段:智能])
#4	#3 AND #2 AND #1

Table S6 Search strategy in CNKI.

Search	Query
#1	主题:"肺肿瘤 + 肺癌 + 肺部癌症 + 肺部肿瘤"
#2	主题:" 静脉血栓栓塞 + 静脉血栓形成 + 深静脉血栓 + 肺栓塞 "
#3	主题:"危险因素"
#4	主题:(#1) AND 主题:(#2) AND 主题:(#3)

Table S7 Search strategy in Wanfang database.

Search	Query
#1	主题:(肺肿瘤肺癌 OR 肺部癌症 OR 肺部肿瘤)
#2	主题:(静脉血栓栓塞 OR 静脉血栓形成 OR 深静脉血栓 OR 肺栓塞)
#3	主题: (危险因素)
#4	主题:(#1) AND 主题:(#2) AND 主题:(#3)

Table S8 Search strategy in VIP.

Search	Query
#1	题名或关键词:(肺肿瘤 OR 肺癌 OR 肺部癌症 OR 肺部肿瘤)
#2	题名或关键词: (静脉血栓栓塞 OR 静脉血栓形成 OR 深静脉血栓 OR 肺栓塞)
#3	题名或关键词: (危险因素)
#4	题名或关键词: (#1) AND (#2) AND (#3)
